# Immunosuppressive Treatment for Lupus Nephritis: Long-Term Results in 178 Patients

**DOI:** 10.1155/2016/7407919

**Published:** 2016-12-05

**Authors:** Elena V. Zakharova, Tatiana A. Makarova, Elena V. Zvonova, Alina M. Anilina, Ekaterina S. Stolyarevich

**Affiliations:** ^1^Nephrology Department, City Botkin Memorial Hospital, Moscow, Russia; ^2^Nephrology Chair, State University of Medicine and Dentistry, Moscow, Russia

## Abstract

Lupus nephritis is one of the most severe Systemic Lupus Erythematosus features, defining treatment modality and prognosis. Our retrospective study, including 178 patients treated for lupus nephritis during 23 years with mostly cyclophosphamide-based initial regimens followed by azathioprine or mycophenolic acid, demonstrates 84.8% of renal response with 19.2% of flares, 15-year patient survival 78.7% and kidney survival 76.3%, and low damage accrual. Both patient and kidney survival significantly differ for subgroups that achieved complete or partial renal response and nonresponders: patient 15-year survival 95% versus 65% versus 35%; kidney 15-year survival 100% versus 58% versus 0%, respectively. 51% (24 out of 47) of patients evaluated at the end of the study period sustained complete renal response; however, only 9 of them had 0 disease activity according to SELENA SLEDAI scale, while 13 patients had scores 2–4 due to the serological abnormalities only. We conclude that (1) initial treatment with cyclophosphamide followed by azathioprine is effective and can be used in agreement with International Guidelines until the evidence for biological treatments benefits becomes available; (2) complete and even partial renal response have positive prognostic value, and failure to achieve renal response negatively influences kidney and patient survival; (3) the validity of complete renal response in SLE is questioned by the absence of conventional definition of SLE remission.

## 1. Introduction

Lupus nephritis (LN) is one of the most severe manifestations of Systemic Lupus Erythematosus (SLE), mainly defining treatment modality and prognosis. Approximately 50% of SLE patients develop LN, which increases the risks for renal failure, cardiovascular disease, and death. Clinical presentation of LN varies from mild asymptomatic proteinuria to severe nephrotic syndrome (NS), hematuria, and renal failure [1, 2]. The pathogenesis of LN has not been clarified so far; however, among a huge variety of autoantibodies involved in SLE tissue damage, LN retains the most extensive group and is triggered by complex autoantibody interactions. Development and progression of LN is regarded as a multistep inflammatory process which is incited by anti-DNA and antinucleosome antibodies, culminating in a self-maintaining inflammatory loop with spreading of glomerular inflammation. In the maintenance of the inflammatory process, proinflammatory antibodies are involved, among which anti-C1q is thought to play a major role [3].

Being one of the major features of SLE, renal disorder is listed in the American College of Rheumatology (ACR) Revised Criteria for Classification of SLE [4]. Pathology evaluation of LN is crucial: according to the EULAR/ERA-EDTA recommendations for the management of adult and pediatric lupus nephritis [5], immunosuppressive treatment should be guided by renal biopsy findings, assessed according to the International Society of Nephrology/Renal Pathology Society 2003 classification [6]. Initial treatment (IT) recommended for patients with class III-IV (±V) LN includes mycophenolic acid (MPA) or low-dose intravenous cyclophosphamide (CY) in combination with glucocorticoids. In patients with adverse clinical or histological features, CY can be prescribed at higher doses, while azathioprine (AZA) is an alternative for milder cases. For patients not responding to MPA or CY, switching from MPA to CY and vice versa or introduction of rituximab should be considered. For pure class V LN, presenting with NS, IT options are MPA, CY, or calcineurin inhibitors (cyclosporine, tacrolimus) in combination with oral glucocorticoids. In patients improving after IT, subsequent treatment (ST) with MPA or AZA is recommended for at least 3 years. Calcineurin inhibitors can be considered for ST in pure class V LN. Hydroxychloroquine is currently recommended for all LN patients. KDIGO Clinical Practice Guideline for Glomerulonephritis [7] provides very similar approaches to the LN management.

According to the Treat-to-Target paradigm, the treatment target in SLE patients should be remission of systemic symptoms and organ manifestations or, if remission cannot be reached, the lowest possible disease activity, measured by a validated lupus activity index and/or by organ-specific markers. Since damage predicts subsequent death, prevention of damage accrual should be a major therapeutic goal in SLE. SELENA SLEDAI Disease Assessment Scale and SLICC/ACR Damage Index are recommended for assessment of SLE activity and damage [8].

Systemic Lupus Erythematosus Disease Activity Index (SLEDAI), modified in the Safety of Estrogens in Lupus Erythematosus National Assessment (SELENA) trial and known as SELENA SLEDAI system, is a list of 24 clinical and laboratory descriptors, scored on the basis of their presence or absence in the previous 10 days before scoring. The maximum theoretical score for the SELENA SLEDAI is 105 (all 24 descriptors present simultaneously) with 0 indicating inactive disease. The Systemic Lupus International Collaborative Clinics/American College of Rheumatology (SLICC/ACR) Damage Index was designed and validated for SLE patients to capture nonreversible organ damage, not related to active inflammation, and lasting at least 6 months [9–11].

However, while remission was used to be described as a favourable clinical state for patients with SLE since at least 1970s, there has not yet been an agreed-upon definition of remission in SLE. There are a number of different ad hoc definitions of remission that have been used in clinical trials and observational studies. The definition of SLE remission, merging clinical disease activity, serological activity, duration, and subsequent treatment still is under discussion [12]. The recent analysis highlights important ongoing disease activity, symptom burden, and immunosuppressive medication in European patients with SLE considered by their treating physician to be “in remission,” indicating that for a further improvement of outcomes there is an urgent need for an international consensus on the definitions for remission among patients with SLE [13].

On the other hand, instruments for lupus nephritis evaluation are currently developed. Although the definitions of remission for LN were controversial for more than two decades [14, 15], and the impact of decrease of proteinuria versus hematuria is not completely clear so far [16], KDIGO, based on the evaluation of published clinical trials, provides definitions for the response to therapy in LN as follows: complete response (CR)—return of serum creatinine (SCr) to previous baseline plus decline in urine protein/creatinine ratio (uPCR) to <50 mg/mmol; partial response (PR)—stabilisation or improvement of SCr but not to normal range, plus ≥50% decrease in uPCR and uPCR ≤300 mg/mmol [7]. EULAR/ERA-EDTA recommendations also point that immunosuppressive treatment targets are complete renal response (proteinuria <0.5 g/24-hr with normal or near-normal renal function) or at least partial renal response (≥50% reduction in proteinuria with decrease to subnephrotic levels and normal or near-normal GFR), which should be achieved preferably by 6 months and no later than 12 months following treatment initiation [4].

In this retrospective study, we aimed to evaluate some demographic and clinical features, pathology patterns, treatment results, and outcomes in the group of patients with lupus nephritis, receiving immunosuppressive treatment in our unit for 23 years, and asses the remission status in the cohort followed till 2015.

## 2. Materials and Methods

### 2.1. Patient's Selection and Workup

Using electronic database and specifically designed charts, we selected 185 SLE patients, treated in our centre in 1992–2015. Workup, beyond routine, included lupus serology tests (anti-DNA antibodies, antinuclear antibodies, anticardiolipin antibodies, lupus anticoagulant, and C3/C4 complement) and kidney biopsy. Diagnosis was based on ACR criteria.

### 2.2. Kidney Biopsy

Kidney core biopsy was taken with BARD-Magnum biopsy guidance facility. Obtained specimens were divided into two parts and processed for light microscopy and immunohistology. Formalin fixed/paraffin embedded sections for light microscopy were stained with hematoxylin and eosin, Masson's trichrome, and periodic acid-Shiff. Unfixed cryosections stained for IgA, IgG, IgM, C3, C1q, kappa and lambda light chains, and fibrinogen. Kidney biopsies were evaluated by dedicated nephropathologists according to ISN/RPS Classification; biopsies obtained before 2004 were reassessed for current analysis.

### 2.3. Treatment Regimens

IT regimens included high dose i.v. and oral steroids in combination with i.v. CY, MPA, cyclosporine-A (CyA), or AZA. Low-dose steroids combined with MPA, AZA, CyA, and i.v. CY quarterly were used for ST. In some patients, steroids only were used both for IT and ST. Hydroxychloroquine was added on top of any regimen since 2012. Anticoagulants and/or antiplatelet agents were used in patients with antiphospholipid syndrome and circulating antiphospholipid antibodies. Rituximab was used as a rescue therapy since 2013 in selected refractory cases.

### 2.4. Results Assessment

Primary efficacy end points, complete response (CR) and partial response (PR), for LN were evaluated according to the degree of proteinuria and SCr level based on KDIGO definition, plus resolving of hematuria. Failure to achieve at least PR in 12 months of IT was considered as no response (NR). “Hard” outcomes were defined as patient's death and kidney death, which was defined as the progression to end stage of renal disease (ESRD).

SELENA SLEDAI Disease Assessment Scales and SLICC/ACR Damage Index were used for SLE activity and damage accrual evaluation.

### 2.5. Statistics

Statistical analysis was performed using SPSS 11.5 program package. Differences significance for categorical variables was evaluated by Fisher's exact test and *χ* ^2^ test. For abnormally distributed variables, median value and interquartile range were calculated, and Mann-Whitney* U* test and Kruskall-Wallis test were used for comparison of these variables. *p* value < 0.05 was defined for statistical significance.

## 3. Results

### 3.1. Study Population

Patients with SLE constituted 1.7% (185 out of 10599) of subjects treated in our nephrology clinic over more than 20 years. Study group included 28 (15.1%) males and 157 (84.8%) females with median age of 29 [15; 70] years; 173 (93.5%) were Caucasian and 12 (6.5%) were Asian. In 89 (48.1%) cases, SLE was first diagnosed in our centre, and 96 (51.9%) patients were referred from other centres, mostly rheumatology, with previously diagnosed SLE.

### 3.2. Clinical Presentation

Patients presented with hematuria, proteinuria/NS, impaired kidney function, and multiple extrarenal manifestations; LN clinical features are shown in Table 1.

### 3.3. Pathology Presentation

108 (58.3%) patients underwent kidney biopsy; in 15 cases (13.8% out of biopsied patients), the second kidney biopsy was performed in 6–118 months after the first biopsy (Table 2).

### 3.4. Immunosuppressive Treatment Regimens

Seven patients did not receive immunosuppressants and were excluded from further analysis. 165 out of 178 patients on immunosuppression were started on IT; 111 patients received ST; 96 patients were treated with both IT and ST in our centre. Treatment regimens are shown in Table 3. Hydroxychloroquine in 51 (28.6%) cases and anticoagulants and/or antiplatelet agents in 83 (46.6%) were used on the top of any regimen.

### 3.5. Initial Treatment Results

CR of LN in 63 (35.3%) cases and PR of LN in 88 (49.4%) cases were achieved, while in 27 (15.1%) patients treatment failed. Among those 151 who achieved remission, 122 (80.7%) sustained remission status and 29 (19.2%) patients subsequently developed renal flares.

### 3.6. Long-Term Outcomes

Median follow-up period comprised 12 [1; 236] months. At the end of the study period (last assessment, December 2015), 47 (26.4%) out of 178 patients on immunosuppression were alive and not on dialysis, 18 (10.1%) started dialysis, 95 (53.3%) were lost for follow-up, and 18 (10.1%) died.

In patients who did not develop ESRD and did not recover kidney function at the last evaluation, median SCr was 182 [115; 580] *μ*mol/L. 32 patients completely recovered kidney function.

Causes of death were thrombotic complications of antiphospholipid syndrome in 7 cases, infectious complications in 5 cases, cardiac failure in 4 cases, and intracranial haemorrhage in 2 cases.

### 3.7. Patient and Kidney Survival

**Figure 1:**
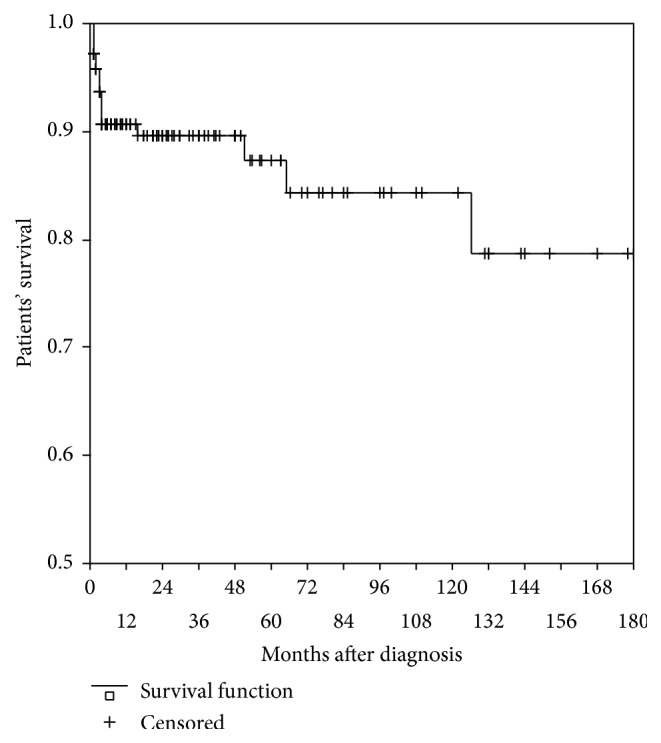
15-year patient survival.

**Figure 2:**
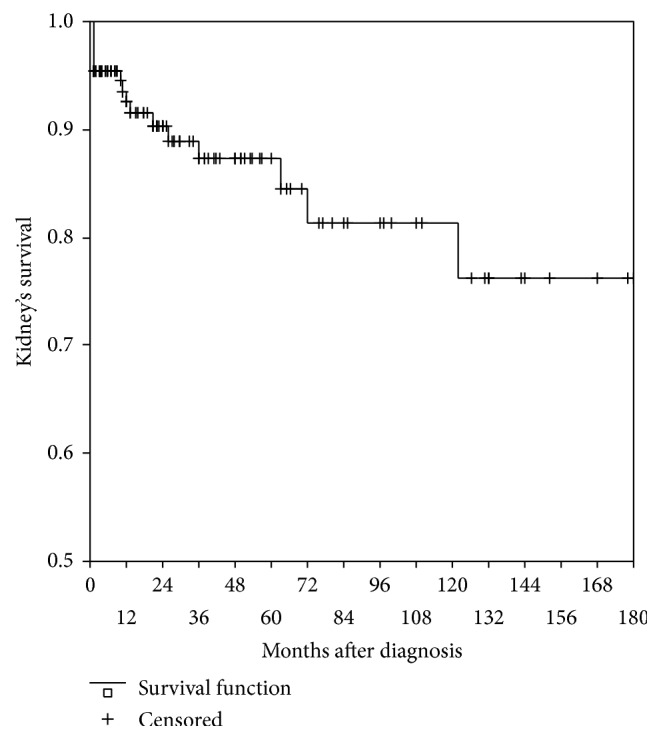
15-year kidney survival.

We did not find differences in the overall patient and kidney survival. 5-year patient and kidney survival were 87.2% and 87.3%, respectively, 10-year patient and kidney survival were 81.3% and 81.4%, respectively, and 15-year patient and kidney survival turned to be 78.7% and 76.3%, respectively, as shown in Figures 1 and 2.

**Figure 3:**
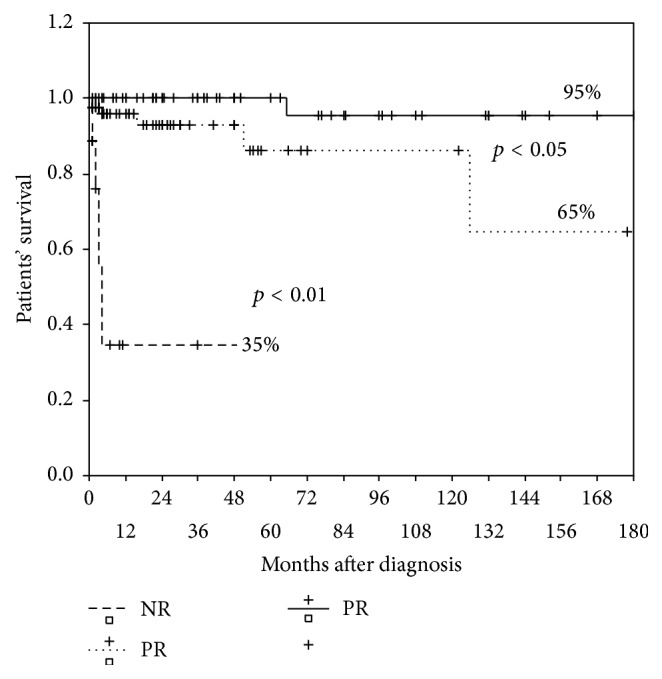
15-year patient survival in patients with CR, PR, and NR.

We analysed patient and kidney survival with respect to CR and PR of LN, achieved after IT, or to NR. 15-year patient survival was 95% for CR of LN versus 65% for PR of LN. In cases with NR, 5-year patient survival was only 35% (Figure 3). All the differences were statistically significant (*p* < 0.01).

**Figure 4:**
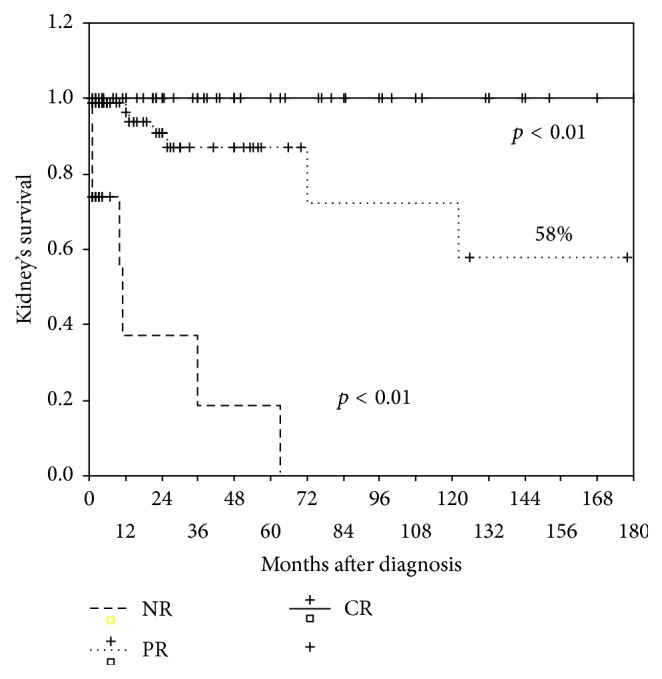
15-year kidney survival in patients with CR, PR, and ND.

15-year kidney survival was 100% in patients with CR of LN versus 58% in patients who achieved only PR of LN. In patients with NR kidney death occurred in all cases to the 5th year of follow-up (Figure 4). All the differences were statistically significant (*p* < 0.01).

### 3.8. Remission Status

47 patients were evaluated for LN remission status, SLE activity, and damage accrual at the latest follow-up visit in 2015. In this cohort, 24 (51.0%) patients achieved and sustained CR, and 21 (44.7%) had PR of LN. Only 2 (4.3%) patients, who previously achieved CR, had a nonresolved renal flare at the latest follow-up assessment. SELENA SLEDAI Disease Assessment Scales and SLICC/ACR Damage Index data for these patients are shown in Table 4.

Among 24 patients with sustained CR of LN, only 9 (37.5%) had score of 0 disease activity, 13 (54.1%) had scores of 2–4, and 2 had score of 6 according to SELENA SLEDAI Disease Assessment Scales. In all 13 cases with CR of LN and SELENA SLEDAI scores 2–4, disease activity presented only by increased anti-DNA antibodies and/or decreased complement levels.

## 4. Discussion

In our LN patients population prevailed young women of Caucasian origin, almost half of them with newly diagnosed SLE, mainly presenting with NS, hematuria, impaired kidney function, and diffuse or focal proliferative LN (classes III and IV) by pathology. Our retrospective study includes patients treated long before International Guidelines, outlining the exclusively low threshold for kidney biopsy indications that were developed [5, 7]; therefore, the proportion of biopsy proven LN is only 58%, reflecting the fact that in early 90s we rarely biopsied patients with less severe clinical manifestations.

Immunosuppressive treatment regimens in our group are compatible with the current guidelines recommendations [5, 7]. Combination of steroids and CY was the dominant treatment option for IT, while MPA and AZA in equal proportion were more often used for ST. The only exception is the usage of steroids only for IT and/or ST in early 90s. That time steroids only were used in patients who did not tolerate or refused CY/AZA and could not receive MPA, which was not available for LN treatment in our country before 1999. Cyclosporine was used for IT and ST mostly in patients with class V LN, which again matches the current guidelines recommendations. We did not analyze rituximab usage results, as it was not available for LN treatment until 2013, and since that it was always second treatment option after IT failure. We also did not analyze hydroxychloroquine and anticoagulants/antiplatelets impact, as that was beyond the scope of the current research.

IT overall efficacy (CR plus PR) turned to be 84.8%, with the rate of CR 35.3%, which is similar to Chen et al. data [15] and higher than the ALMS study [17], probably because our study group included not only patients with LN lass III–V but also milder cases. Under ST, the rate of flares turned to be 19.2% during median follow-up of 12 [1; 236] months, similar to the data from the long-term follow-up of the MAINTAIN Nephritis Trial [18]. We did not specifically address the issue of different immunosuppression regimens efficacy in this study, but the general clinical assessment does not suggest benefits of MPA over CY and AZA in our group of patients, which is in agreement with the findings from ALMS study and long-term follow-up of the MAINTAIN Nephritis Trial [17, 18].

Patient and kidney overall 15-year survival were higher than 75%. Importantly, in those who achieved CR after IT, patient and kidney 15-year survival were 95% and 100%, respectively. In patients who achieved PR, patient and kidney survival were 65% and 58%, respectively, and in nonresponders they were 35% and 0%, respectively. These differences confirm the positive prognostic value of complete and even partial LN response [15, 16], associated with significantly better outcomes compared to NR, and stress that failure to achieve renal response to immunosuppression negatively influences not only kidney but also patient survival.

Almost half of biopsy proven LN cases were available for evaluation at the end of the study period. Number of remissions increased to 95.7%, confirming the higher efficacy of biopsy-guided treatment [5, 7].

In terms of remission assessment, it is important to highlight that among 24 patients with sustained CR of LN more than a half had scores 2–4 by SELENA SLEDAI Disease Assessment Scales due to the elevated anti-DNA antibodies and complement abnormalities. These data support the need for the agreed-upon definition of remission in SLE [12].

Damage accrual was relatively low; majority of patients had scores 0–2 according to SLICC/ACR Damage Index, mostly due to steroid cataract, diabetes, osteoporosis, or incomplete recovery of kidney function. Steroid therapy complications clearly prevailed, confirming the necessity of tapering or even discontinuation of steroid usage after 3 years of sustained remission [5, 7].

## 5. Conclusions

Treatment results and long-term outcomes in our group of 178 lupus nephritis patients, treated during the 23-year period with mostly cyclophosphamide-based initial regimens followed by azathioprine or mycophenolic acid, demonstrate 84.8% of renal response with only 19.2% of flares during 12 [1; 236] months of follow-up, overall 15-year patient and kidney survival of 78.7% and 76.3%, respectively, and low damage accrual. We conclude that initial treatment with cyclophosphamide and subsequent treatment with azathioprine ensure high efficacy and good safety profile and can be used according to current International Guidelines until the evidence for biological treatments benefits becomes available.

Patient and kidney survival significantly differed between subgroups that achieved complete renal response, partial renal response, and nonresponders, with patient 15-year survival 95% versus 65% versus 35%, respectively (*p* < 0.01), and kidney 15-year survival 100% versus 58% versus 0%, respectively (*p* < 0.01). We conclude that complete and even partial renal response has a positive prognostic value, while failure to achieve renal response to immunosuppression negatively influences not only kidney's but also patients' survival.

In the cohort of 47 patients followed up at the end of the study period, 51% demonstrated sustained complete renal response. However, only 9 out of these 24 patients had 0 disease activity according to SELENA SLEDAI Disease Assessment Scale, while 13 patients had scores 2–4 due to the elevated anti-DNA antibodies and complement abnormalities without clinical activity features. We conclude that the validity of complete renal response in SLE is questioned by the absence of conventional definition of SLE remission and the uncertain value of serological abnormalities.

## Figures and Tables

**Table 1:** Clinical presentation of lupus nephritis.

Symptom	Haematuria	Proteinuria	Nephrotic syndrome	Impaired kidney function	SCr in patients with impaired kidney function, *μ*mol/L
*n*	161	95	90	92	236 [121; 2097]
%	87.0	51.3	48.6	49.7	

**Table 2:** The distribution of lupus nephritis pathology classes.

LN classes	1st biopsy		2nd biopsy	
	*n*	%	*n*	%
Class I	5	4.6	1	6.6
Class II	12	11.1	2	13.3
Class III	22	20.3	3	20
Class IV	42	38.8	3	20
Class V	15	13.8	2	13.3
Class V + class III/IV	3	2.7	2	13.3
Class VI	9	8.3	2	13.3
Total	108	100	15	100

**Table 3:** Treatment regimens for initial and subsequent therapy.

	Steroids + CY		Steroids + MPA		Steroids + CyA		Steroids + AZA		Steroids only		Total
	*n*	%	*n*	%	*n*	%	*n*	%	*n*	%	
IT	90	54.5	11	6.6	20	12.1	20	12.1	24	14.5	165
ST	5	4.5	27	24.3	17	15.3	30	27.0	32	28.2	111

**Table 4:** SELENA SLEDAI and SLICC scoring in the cohort of 47 patients with LN remission.

	SELENA SLEDAI				SLICC/ACR			
	0	2–4	6–8	10–12	0	1-2	3-4	5-6
*n*	19	18	8	2	14	20	11	2
%	40.4	38.2	17.0	4.2	29.7	42.5	23.4	4.2
